# Anatomical study of the proximal tibiofibular ligaments using ultrasound

**DOI:** 10.1186/s13244-021-00965-z

**Published:** 2021-02-18

**Authors:** Laura Scarciolla, Matthias Herteleer, Edouard Turquet, Sammy Badr, Xavier Demondion, Thibaut Jacques, Anne Cotten

**Affiliations:** 1 Service de Radiologie et Imagerie Musculosquelettique, CCIAL, CHU de Lille, 59037 Lille, France; 2 Laboratoire d’Anatomie, Faculté de Médecine, Université de Lille, Lille, France; 3 Unité de Taphonomie Médico-Légale et Anatomie (UTML & A), EA 7367, Université de Lille, Lille, France; 4 Faculté de Médecine, Université de Lille, Lille, France

**Keywords:** Anatomy, Articular ligaments, Instability, Joint, Knee, Ultrasound

## Abstract

**Objectives:**

No description of the proximal tibiofibular (PTF) ligaments by means of high ultrasound has yet been reported in the literature. The purpose of this study was to assess whether ultrasound may allow the assessment of these ligaments.

**Methods:**

This study was initially undertaken in three cadaveric knees, followed by an ultrasound study performed by two musculoskeletal radiologists working in consensus of 52 patients without history of trauma or surgery of the knee, and without lateral knee pain. The visibility, echogenicity, length and thickness of the PTF ligaments were assessed.

**Results:**

Regarding the anterior PTF ligament, the superior bundle and the upper and lower middle bundles were clearly seen in 42.3%, 100% and 75% of the knees, respectively. Regarding the posterior PTF ligament, the superior and middle bundles were clearly seen in 88.4% and 51.9% of the knees, respectively. The echo-anatomy of these ligaments and the probe positioning allowing their best depiction were described in this study.

**Conclusion:**

Most of the PTF ligaments can be visualized by means of ultrasound. This possible assessment may have clinical applications, particularly in patients with lateral knee pain.

## Key points

- Most of the proximal tibiofibular (PTF) ligaments can be visualized by means of ultrasound.
- Precise knowledge of the anatomy of these articular ligaments is important for their depiction by means of ultrasound.
- Ultrasound assessment of the PTF ligaments might be useful in patients with chronic instability of the PTF joint or with lateral knee pain.

## Introduction

The proximal tibiofibular (PTF) joint has been called the “forgotten joint” in the literature [1] as its anatomy and pathology frequently receive little attention. This joint is stabilized by thick and strong anterior ligaments and by thinner posterior ligaments [2]. PTF joint instability includes a wide spectrum of clinical manifestations, ranging from acute dislocation to chronic instability [3]. In the latter case, the symptoms can be subtle and non-specific, which explains why these patients are often overlooked and misdiagnosed [4]. Moreover, there is a paucity of literature detailing the specific imaging features associated with chronic instability [5]. The latter disorder includes thickening and partial-thickness tear of the PTF ligaments on MR images [3].

In our department, the PTF joint is systematically assessed with ultrasound. However, no clear standardization of the US protocol exists among our radiologists regarding the PTF ligaments, and none is available in the literature, as, to the best of our knowledge, no description of the PTF ligaments by means of ultrasound has yet been reported. The purpose of our study was to assess whether ultrasound may allow for the assessment of the PTF ligaments.

## Materials and methods

### Anatomical study on cadavers

The study was initially undertaken on two human non-paired cadaveric lower limbs (one male and one female) in order to gain a better understanding of the anatomy of the proximal tibiofibular (PTF) ligaments. The cadavers were donated for the purposes of research and education of human anatomy. They were embalmed using a preparation which included distilled water, glycerine, methanol and phenol, which allowed for the preservation of the consistency of the tissue and the range of joint motion. None of them presented a history of prior injury and/or surgery of the knee. Both dissections were performed by a trained anatomist.

Skin and subcutaneous tissue were removed, exposing the underlying fascia and iliotibial band. Laterally, removal of fascia allowed for the resection of the distal attachments of the *biceps femoris* tendon including its anterior arm. Anteriorly and laterally, proximal attachments of the leg muscles were also removed. Posteriorly, muscles were resected leaving only the popliteus tendon which was then cut at one extremity and reflected. Secondly, the lateral collateral ligament was removed so as to better see the underlying ligaments and capsular structures. Careful dissection was then undertaken to identify the anterior and posterior ligaments of the PTF joint and the relevant bony landmarks. Their length and thickness were also measured.

A third knee was then used to confirm the accuracy of the correct depiction of the PTF ligaments using ultrasound. The target ligaments were transfixed by two musculoskeletal radiologists, in consensus, under continuous ultrasound control, using one Ethicon Vicryl ® 3/0 stitch (19 mm reverse cutting needle). A 17LH7 MHz linear imaging probe (Aplio i450 ultrasound device, Canon Medical Systems) was used for this procedure. A subsequent dissection of the knee was then performed as described above, to ensure that the ultrasound target corresponded to the anatomical target.

### Patients and ultrasound technique

Between August 2019 and February 2020, 52 patients (14 females and 38 males, median age of 34 years, (min 20 years, max 56 years) were included in this study, which was conducted in in the Musculoskeletal Imaging Department of the University Hospital of Lille (France). Inclusion criteria were patients who had been addressed to our department for a knee ultrasound examination (patients addressed for patellar or quadriceps tendinopathy, posterior pain including popliteal cysts, medial pain including bursitis and degenerative changes), performed in clinical routine in consensus by the same two radiologists (a senior musculoskeletal radiologist and a radiology resident). Exclusion criteria were patients under the age of 18 years old, history of trauma or surgery of the knee, lateral knee pain and incomplete ultrasound examination protocol. Each patient provided informed consent. This study was declared and approved by the institutional board under the number CRM-1907-023.

All the ultrasound examinations were performed using an Aplio i800, (Canon Medical Systems) with a high-frequency linear transducer (18Lx5 MHz), except for the middle bundle of the posterior complex, for which a lower frequency transducer (14Lx5 MHz) was also used due to ligament depth.

**Fig. 1:**
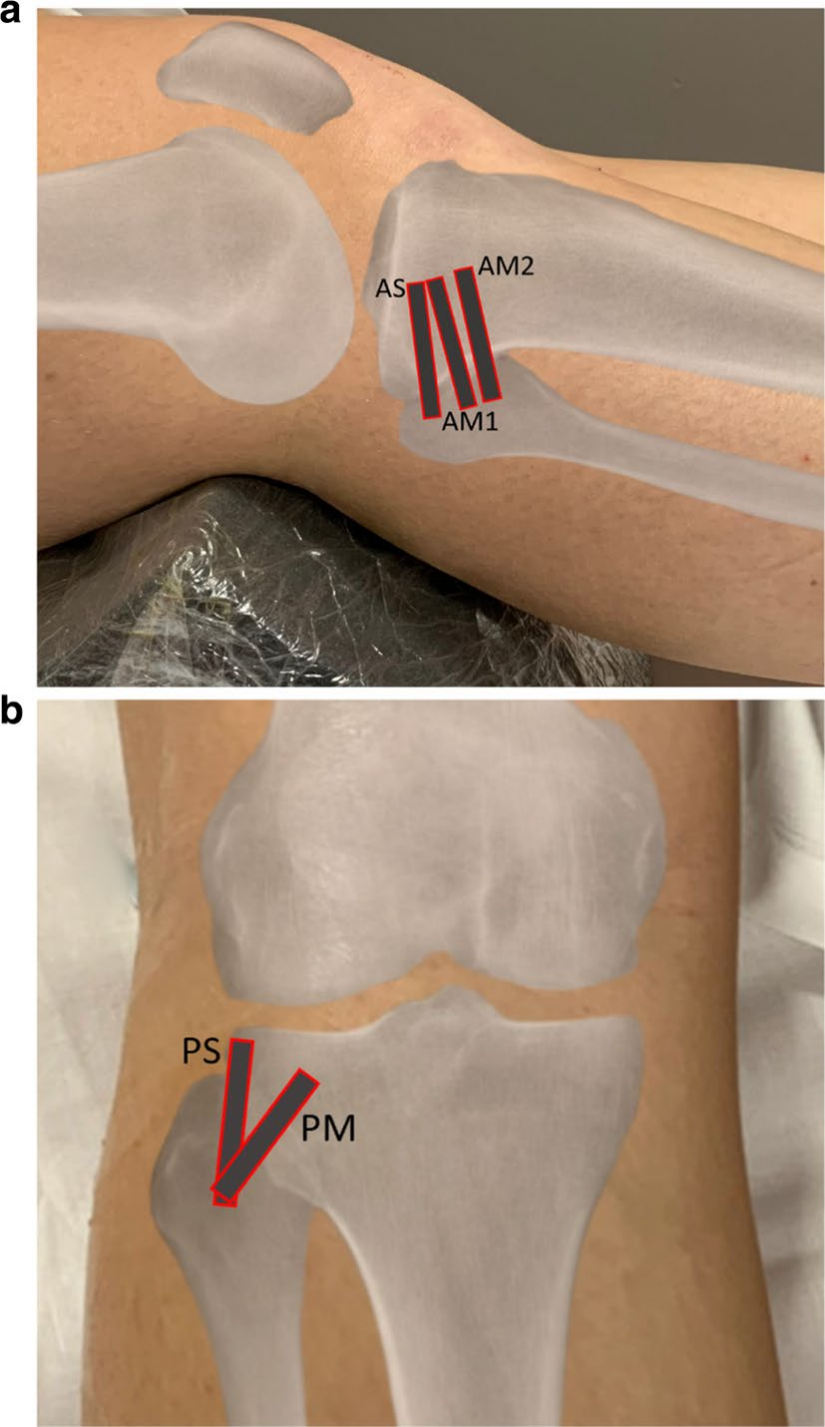
Probe and patient positions for the assessment of anterior PTF ligaments. **a** AS: superior bundle, AM1: upper middle bundle; AM2: lower middle bundle. **b** PS: superior bundle; PM: middle bundle

Fifty-two knees (27 right / 25 left) were analyzed. Patients were examined in the supine position with a 20°–30° of knee flexion for the assessment of the different anterior PTF bundles (Fig. 1a) and in prone position, with the knee extended for the posterior PTF bundles (Fig. 1b). For each bundle, particular attention was paid to keeping the beam parallel to the long axis of the ligament to avoid anisotropic artefacts.

The visibility of the PTF ligaments was classified either as good (ligament nicely demonstrated in its length, including at both insertions) or poor. The echogenicity of the bundles, their length and thickness and their bony attachments were assessed. Differentiation of the superior bundle of the anterior ligament from the overlying anterior arm of *biceps femoris* tendon was also analyzed and classified as good (separation between the two structures) or absent (no clear separation). Colour Doppler was used in each examination in order to detect vascular structures adjacent to the anterior PTF ligaments.

## Results

### Anatomical study on cadavers

**Fig. 2:**
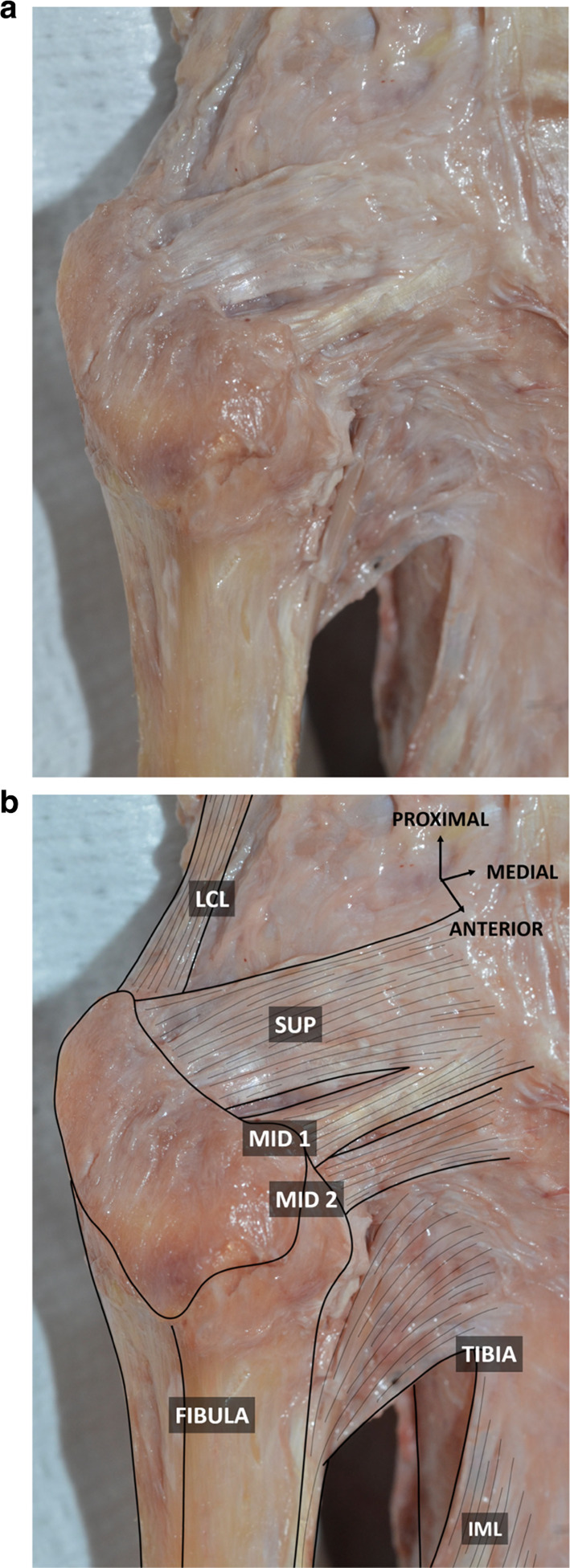
Anterolateral view of the proximal tibiofibular joint without (**a**) and with (**b**) annotations. Muscular structures have been removed, exposing the underlying anterior PTF ligament. SUP: superior bundle of the anterior PTF ligament; MID 1: upper middle bundle of the anterior PTF ligament; MID 2: lower middle bundle of the anterior PTF ligament. LCL: lateral collateral ligament; IML: interosseous membrane of the leg

In each specimen, the anterior PTF ligament consisted of three flat bands (Fig. 2): one superior and 2 middle bundles. The superior bundle (length: 15.1 and 16 mm) was inserted on the anterolateral aspect of the styloid process and the adjacent fibular head, just anterior and slightly inferior to the attachment of the anterior arm of the *biceps femoris* tendon. It coursed slightly upwards and forwards to insert on the lateral aspect of the tibia, just posterior to the attachment of the anterior arm. The upper middle bundle (M1) (length: 12.7 and 14 mm) extended from the anterosuperior aspect of the lateral edge of the fibular head to the lateral aspect of the tibia, inserting just distally to the tibial attachment of the superior bundle. The lower middle bundle (M2) (length: 7 and 9.2 mm) was identified below and deeper than the previous one due to its insertion on the anterior aspect of the fibular head. No inferior bundle could be identified.

**Fig. 3:**
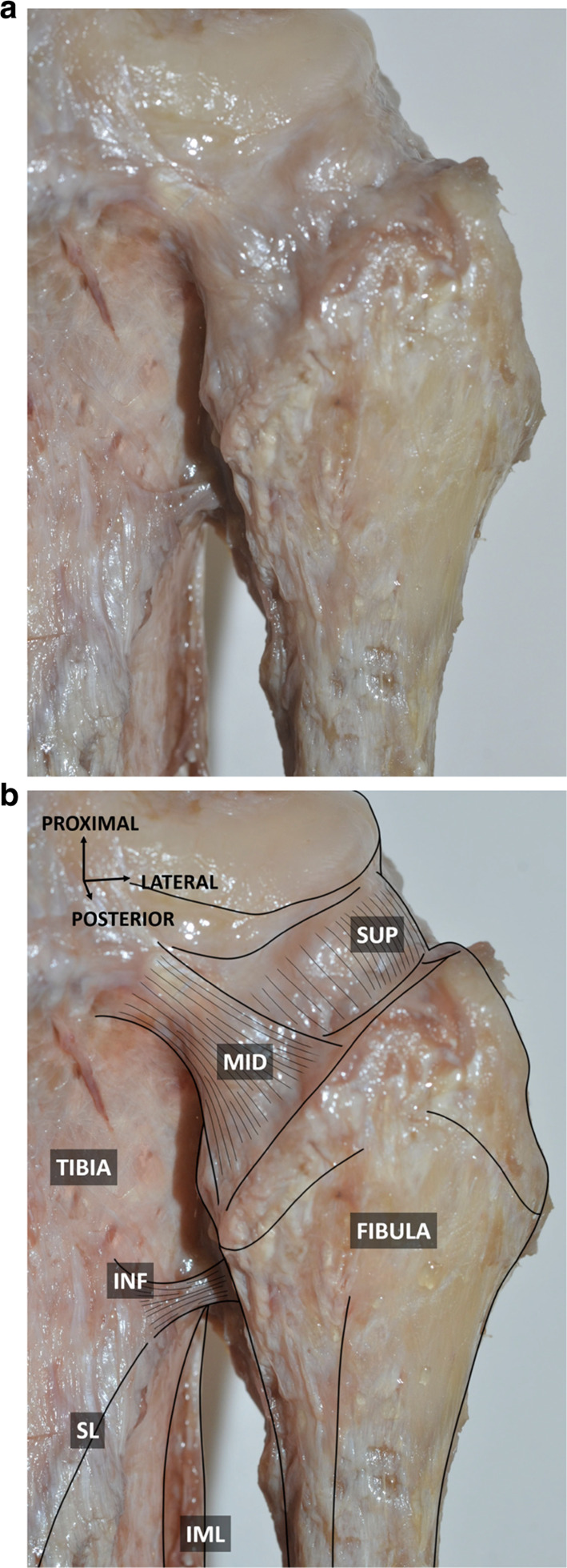
Posterior view of the proximal tibiofibular joint without (**a**) and with (**b**) annotations. Muscular structures and lateral collateral and popliteofibular ligaments have been removed. The superior bundle can be seen as a thickening of the PTF joint capsule. The inferior bundle can be seen as a reinforcement emanating from the soleus muscle. SUP: superior bundle of the posterior PTF ligament; MID: middle bundle of the posterior PTF ligament; INF: inferior bundle of the posterior PTF ligament; SL: soleal line; IML: interosseous membrane of leg

Regarding the posterior PTF ligament (Fig. 3), the superior bundle appeared as a reinforcement of the PTF joint capsule, as more than just a distinct ligament (length: 3.7 and 4.1 mm). It was inserted on the posterosuperior aspect of the styloid process, medially to the popliteofibular ligament. It extended upwards to the posterior aspect of the lateral tibial condyle. The middle bundle was seen as a ligamentous structure (length: 9.1 and 10.2 mm). It extended upwards and medially from the posterior and medial aspect of the fibular head to the adjacent tibia. The inferior bundle appeared as a reinforcement emanating from the soleus muscle. It was identified between the fibular neck and the adjacent tibia, inserting just superior to the soleal line.

The dissection of the third knee confirmed that the PTF ligaments (3 bundles anteriorly and 2 posteriorly) were correctly transfixed. No inferior bundle was identified and was therefore not able to be transfixed using ultrasound.

### Patients and ultrasound technique

#### Anterior PTF ligaments

To assess the superior bundle, an initial depiction of the anterior arm of the *biceps femoris* tendon insertion was found to be useful, due to the close proximity between the two structures. This anterior arm was easily recognized on axial images by its continuity with the *biceps femoris* tendon just proximal to its insertion on the styloid process.

**Fig. 4:**
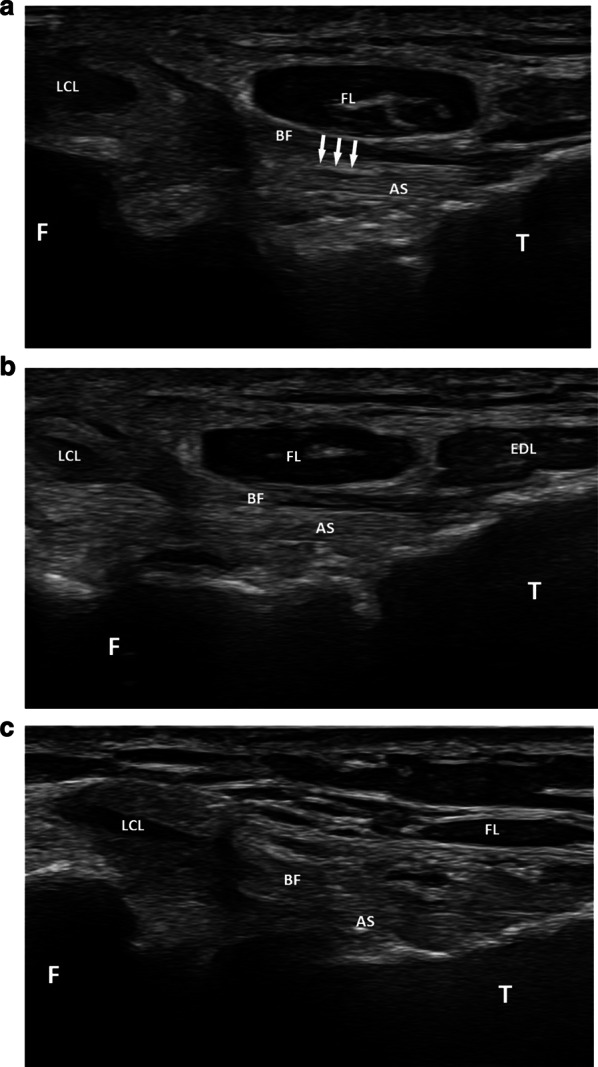
Superior bundle of the anterior PTF ligament (transverse plane). **a** The superior bundle (AS) is well differentiated from the more superficial anterior arm of the *biceps femoris* muscle (BF). It extends from the anterior aspect of the styloid process and the adjacent fibular head (F) to the lateral aspect of the tibia (T). Note the hyperechoic space between them (arrows). **b** Close proximity between the superior bundle (AS) and the anterior arm (BF) but their different anisotropy allows their differentiation. **c** Close proximity between the superior bundle (AS) and the anterior arm (BF) but no clear differentiation between them due to similar anisotropy. LCL: lateral collateral ligament; FL: *fibularis longus* muscle; EDL: *extensor digitorum longus* muscle

In 22 patients (42.3% of the cases), the differentiation between the superior bundle and the anterior arm was good, with a thin hyperechoic band seen between them (Fig. 4). The superior bundle was seen as a hyperechoic and thin structure on a transverse plane oriented slightly upwards (0°–10°) and forwards. It extended from the anterior aspect of the styloid process and the adjacent fibular head to the lateral aspect of the tibia, approximately 2 cm posteriorly to Gerdy’s tubercle. Its echogenicity was similar to the one of the adjacent anterior arm. Its mean length and thickness were 15.98 mm (SD = 1.60) and 1.24 mm (SD = 0.20), respectively, (Table 1). In the other 30 patients (57.7% of the cases), the superior bundle leaned against the anterior arm without any hyperechoic space between them, making the differentiation between these two structures more difficult (Fig. 4).

**Table 1:** Length and thickness of the PTF ligaments. *PTF* proximal tibiofibular, *SD* standard deviation, *mm* millimetre

Bundle	Length (mm)			Thickness (mm)		
	Mean	SD	Range	Mean	SD	Range
*Anterior PTF ligament*						
Superior	15.98	1.60	12.4–19.2	1.24	0.20	0.7–1.8
Upper middle (M1)	12.34	0.90	10.6–14.7	1.21	0.19	0.7–1.6
Lower middle (M2)	8.20	1.12	6.3–10.9	1.16	0.23	0.8–1.7
*Posterior PTF ligament*						
Superior	4.12	0.68	2.9–6	0.88	0.19	0.7–1.3
Middle	8.12	0.90	6.2–10	1.89	0.46	0.9–2.8

**Fig. 5:**
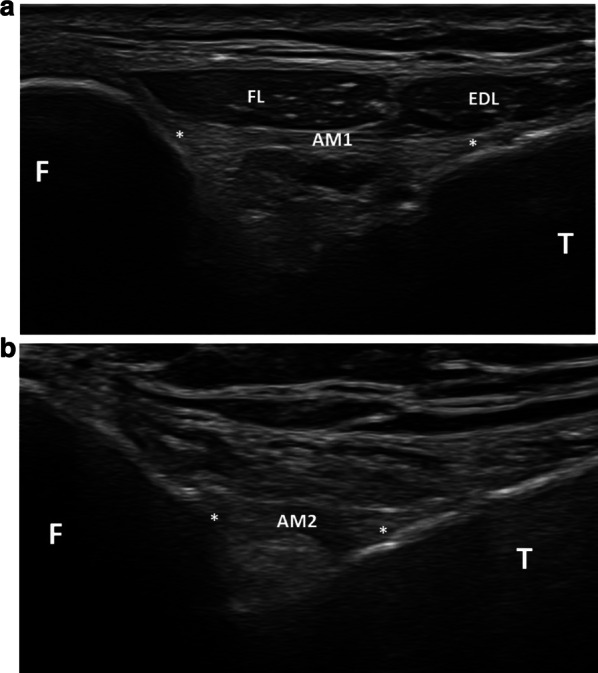
Middle bundles of the anterior PTF joint. **a** Transverse plane showing the upper middle bundle (AM1 and stars), which courses from the anterosuperior aspect of the lateral edge of the fibular head (F) to the lateral aspect of the tibia (T). FL: *fibularis longus* muscle; EDL: *extensor digitorum longus* muscle. **b** Slightly upwards and forwards oblique transverse plane showing the lower middle bundle (AM2 and stars) extending from the anterior aspect of the fibular head (F) to the lateral edge of the tibia (T)

The upper middle bundle (M1) was able to be detected as a hyperechoic structure in each patient (Fig. 5). It coursed on a transverse plane (0°–10° upwards) below the superior bundle from the anterosuperior aspect of the lateral edge of the fibular head to the lateral aspect of the tibia approximately 2 cm posteriorly to Gerdy's tubercle. Its mean length and thickness were 12.34 mm (SD = 0.90) and 1.21 mm (SD = 0.19), respectively.

The lower middle bundle (M2) was able to be identified in 39 patients (75% of the cases) (Fig. 5). This hyperechoic ligament extended below the upper middle bundle in an upward (20°–30°) and forward oblique plane, from the anterior aspect of the fibular head to the lateral edge of the tibia. It showed a common tibial insertion with M1 but in the region of its fibular attachment, a thin (1–2 mm) hyperechoic linear space was seen in every instance between M1 and M2. One or several venous spots could be identified in this space on power Doppler in 15 knees out of 39 (38.5% of the cases). M2’s mean length and thickness were 8.20 mm (SD = 1.12) and 1.16 mm (SD = 0.23), respectively. M2 was partly seen in 7 patients (13.5% of the cases) with a bad depiction of its fibular (4 cases) or tibial (3 cases) insertion. It was not able to be depicted at all in 6 patients (11.5% of cases). No inferior bundle could be identified.

#### Posterior PTF ligaments

**Fig. 6:**
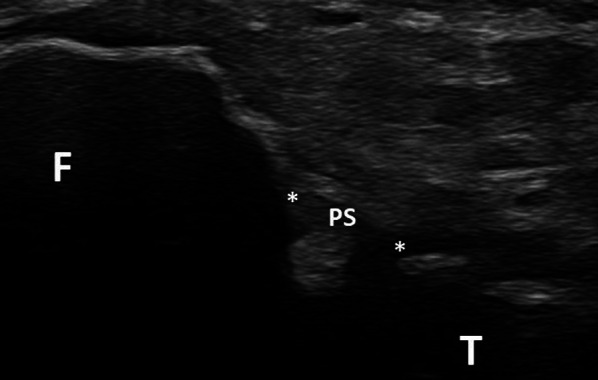
Superior bundle of the posterior PTF ligament (sagittal plane). The superior bundle (PS) courses from the posterior and superior aspect of the fibular styloid (F) to the posterior aspect of the lateral condyle of the tibia (T)

The superior bundle was detected in 46 patients (88.4% of the cases) (Fig. 6). This hypoechoic and slightly convex structure coursed obliquely upwards on a sagittal plane from the posterior and superior aspect of the fibular styloid to the posterior aspect of the lateral condyle of the tibia, overlying the PTF joint.

Its mean length and thickness were 4.12 mm (SD = 0.68) and 0.88 mm (SD = 0.19), respectively.

**Fig. 7:**
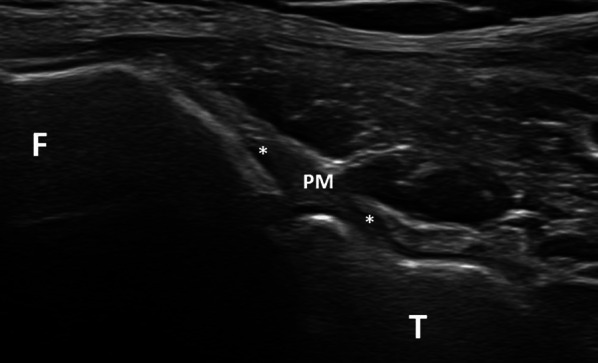
Middle bundle of the posterior PTF joint (oblique and upwards plane). This bundle (PM) courses from the posterior and medial aspect of the fibular head (F) to the adjacent facet of the lateral tibial condyle (T)

The detection of the middle bundle was good in 27 patients (51.9% of the patients) (Fig. 7). This hypoechoic structure coursed on an oblique and upwards plane (40°–50° angle) from the posterior and medial aspect of the fibular head to the adjacent facet of the lateral tibial condyle. Its mean length and thickness were 8.12 mm (SD = 0.90) and 1.89 mm (SD = 0.46), respectively. In 8 patients (15.4% of the cases), only its fibular insertion could be depicted.

No inferior bundle could be identified in our study.

## Discussion

To the best of our knowledge, this is the first ultrasound study assessing the proximal tibiofibular ligaments. This is all the more surprising since this joint is relatively superficial and therefore theoretically accessible to this imaging modality.

Understanding the anatomy is fundamental in the depiction of ligaments using ultrasound. In this regard, there are limited data in the literature describing the anatomy of the PTF ligaments. Moreover, variations in the number, size and orientation of the bundles have been reported.

### Anterior PTF ligaments

Up to four bundles have been described anteriorly [1]. Our cadaveric dissections showed three flat bands anteriorly, one superior and 2 middle bundles. In the literature, the superior bundle seems to be constant, whereas anatomical variations mainly involve the other bundles [1].

When assessing the superior bundle of the anterior PTF ligament with ultrasound, it was found to be useful to start with the assessment of the anterior arm of the *biceps femoris* tendon as this structure was more superficial than the adjacent superior bundle, and consequently easier to depict. In 42.3% of the cases, the superior bundle could be clearly detected as a hyperechoic structure extending from the anterior aspect of the styloid process and the adjacent fibular head to the lateral aspect of the tibia. It was well differentiated from the adjacent anterior arm by a thin echoic band representing fatty tissue. The mean length and thickness of this superior ligament were in accordance with those reported in a cadaveric study [1]. However, in 57.7% of the cases, the anterior arm and the superior bundle were closely applied, making their differentiation difficult. Close proximity and even fusion between them has indeed been described in cadaveric studies [6, 7]. Slight tilting and angulation of the probe was found helpful to depict mild differences in echogenicity due to anisotropy of these two structures in certain cases (Fig. 4).

The upper middle bundle M1 could be seen in all cases as a thin hyperechoic structure extending from the anterosuperior aspect of the lateral edge of the fibular head to the lateral aspect of the tibia. In contrast to our cadaveric study which showed two middle bundles in both specimens, the lower middle bundle M2 was able to be depicted on ultrasound in only 75% of the cases. Differentiation between M1 and M2 was facilitated by the depiction of a hyperechoic linear space between them, which could contain small venous dots. The absence of depiction of M2 in 25% of the cases may be related to its depth, which can make it more difficult to detect than M1 on ultrasound, or to the close proximity between M1 and M2, which can prevent their differentiation when using ultrasound. In fact, M2 has been reported as inconstant in cadaveric studies [1, 8] and some authors have preferred the expression “middle bundle complex” with rare descriptions of a bifurcation of this complex or presence of a deep ligament in this area [1].

No inferior bundle could be detected with ultrasound. This bundle has been reported in only one cadaveric study in 6 out of 10 cases [1]. Its absence, thinness or deep location might explain its non-detection with ultrasound in our study. Moreover, some authors describe a small aberrant bundle, reinforcing the superior part of the interosseous membrane of the leg [9]. This bundle, also known as Barkow’s ligament, could be mistaken as an inferior bundle of the anterior PTF complex, but its relationship with the PTF joint is not clear [9].

### Posterior PTF ligaments

Posteriorly, the anatomy of the PTF ligaments has rarely been reported, with descriptions ranging from three distinct bundles [1] to capsular thickening and no bundles [10]. Whatever their description, the posterior PTF ligaments are considered weaker than the anterior ones [2].

Regarding the superior bundle, its appearance on the cadavers used in our study was more like a capsular thickening of the PTF joint than a distinct ligament. This ligament has also previously been reported as a focal capsular thickening in cadavers studied with histology, MRI and MR arthrography [11]. It separates the PTF joint from the subpopliteal recess. This structure was detected on ultrasound in 88.4% of the cases. It was depicted in a sagittal plane as a hypoechoic structure running from the posterior and superior aspect of the fibular styloid to the posterior aspect of the lateral condyle of the tibia. Its hypoechoic appearance is most likely explained by the obliquity of the probe with regard to the ligament, and by a slight convexity of the latter. The fact that this ligament is in close proximity to the adjacent bones might explain the absence of depiction of this ligament in 11.6% of the cases. This might also be explained by the presence of a defect within this structure. It is well known that such a defect allows for the communication between the PTF joint and the knee joint during knee arthrography [11].

The middle bundle was clearly identified as a true ligament in our cadavers. It was seen as a hypoechoic structure coursing from the posterior and medial aspect of the fibular head to the adjacent facet of the lateral tibial condyle on ultrasound. However, it was detected in only 51.9% of our patients, probably because of its deep location with juxtaposition of muscles responsible for ultrasound attenuation. Its deep location probably also explains its hypoechogenicity.

We acknowledge several limitations in our study. First, only three cadavers were dissected. However, the goal of our study was not to assess the prevalence of anatomical variations of the PTF ligaments. Second, only 52 patients were included in our study. Larger studies might demonstrate different prevalences of the detection of the bundles. Third, intra- and interobserver reproducibility was not assessed, as all the ultrasound examinations were analyzed in consensus by two musculoskeletal radiologists. This may influence the prevalence of the detection of the PTF bundles. However, the main goal of this study was to describe the ultrasound appearance of these structures. Fourth, we did not correlate the length and thickness of our bundles to measurements of the length of the leg or to the PTF joint morphology. However, this also has not been previously assessed, either on cadaveric or MRI studies. Finally, we did not assess whether the appearance of the bundles was symmetrical. Such an assessment might be useful for the assessment of these ligaments in clinical practice.

## Conclusion

In conclusion, our study has demonstrated that most of the PTF ligaments can be visualized by means of ultrasound. The potential applications of this possible assessment must now be confirmed by clinical studies, particularly in patients with lateral knee pain.

## Abbreviation

PTF: Proximal tibiofibular

## Availability of data and material

All data are published in the article.
